# High-Throughput Sequencing-Based Assessment of Intestinal Parasitic Infections in Economically and Medicinally Valuable Captive Tokay Gecko (*Gekko gecko*) and Chinese Blue-Tailed Skink (*Plestiodon chinensis*)

**DOI:** 10.3390/ani15223298

**Published:** 2025-11-15

**Authors:** Zichao Yu, Yi Xiong, Guanping Xie, Zhengjun Wu

**Affiliations:** 1 Key Laboratory of Ecology of Rare and Endangered Species and Environmental Protection, Guangxi Normal University, Ministry of Education, Guilin 541004, China; 2 Guangxi Key Laboratory of Rare and Endangered Animal Ecology, College of Life Science, Guangxi Normal University, Guilin 541004, China

**Keywords:** captive reptiles, *Gekko gecko*, high-throughput sequencing, intestinal parasites, *Plestiodon chinensis*

## Abstract

Captive reptiles bred for economic and medicinal purposes are often susceptible to intestinal parasites that undermine their health and overall farm productivity. In many regions, their economic value largely derives from medicinal use; accordingly, they are described as reptiles with medicinally driven economic value. In this study, we applied high-throughput sequencing to characterize gut eukaryotic communities and assess parasite prevalence in tokay geckos (*Gekko gecko*) and Chinese blue-tailed skinks (*Plestiodon chinensis*). Across both host species, six pathogenic genera were identified: *Cryptosporidium* (*P. chinensis*: 57.1%, 12/21; *G. gecko*: 0%), *Eimeria* (*P. chinensis*: 14.3%, 3/21; *G. gecko*: 0%), *Oswaldofilaria* (*P. chinensis*: 0%, *G. gecko*: 4.8%, 1/21), *Strongyloides* (*P. chinensis*: 14.3%, 3/21; *G. gecko*: 19.1%, 4/21), *Spironucleus* (*P. chinensis*: 14.3%, 3/21; *G. gecko*: 9.5%, 2/21), *Spauligodon* (*P. chinensis*: 0%, *G. gecko:* 14.3%, 3/21). Among these, *Cryptosporidium* exhibited a striking host-specific pattern, occurring exclusively in Chinese blue-tailed skinks (Fisher’s exact test, *p* = 5.32 × 10^−5^). Principal component analysis (PCA) demonstrated clear host-associated segregation along PC1, which accounted for 58.2% of total variance. Furthermore, co-occurrence network analysis revealed significant positive associations (all *p* < 0.01) between parasites and other eukaryotes, particularly fungi and protozoa. Collectively, these findings underscore the value of high-throughput sequencing for parasite surveillance in reptile farming and point to the importance of integrated diagnostic approaches and enhanced biosecurity measures for maintaining animal health.

## Simple Summary

Captive reptiles raised for economic and medicinal purposes are frequently exposed to intestinal parasites, which can compromise their health and reduce reproductive success. Because their economic value largely derives from medicinal use, they are described as reptiles with medicinally driven economic value. In this study, we examined fecal samples from two commercially farmed species, the tokay gecko (*Gekko gecko*) and the Chinese blue-tailed skink (*Plestiodon chinensis*), using high-throughput sequencing to profile their intestinal eukaryotic communities and identify pathogenic parasitic genera. The overall parasite infection proportion was 57.1% (12/21) in Chinese blue-tailed skinks and 42.9% (9/21) in tokay geckos. Notably, *Cryptosporidium* was detected exclusively in Chinese blue-tailed skinks, with its prevalence showing a highly significant difference between the two hosts (*p* = 5.32 × 10^−5^, Fisher’s exact test). Certain parasites displayed positive associations with fungi and other gut microorganisms, suggesting potential ecological interactions that may influence the course of the infections. Together, these findings highlight the host-specific nature of parasite infection patterns and underscore the importance of monitoring parasite dynamics as part of health management strategies in reptile breeding systems.

## 1. Introduction

Captive production of economically valued animals is expanding rapidly, and effective health management has become a primary constraint on sustainable growth [1]. Within intensive systems, intestinal parasitism is a consistent pressure but is often missed by routine microscopy and screening based on clinical signs, which do not reliably detect mixed or low-intensity infections [2,3]. This underdetection leaves a practical surveillance gap in high-density enclosures, permitting subclinical persistence and raising the likelihood of outbreaks.

Reptiles with both medicinal and economic importance are increasingly reared in captivity to meet the rising demands of traditional medicine and the wildlife trade [4,5]. Many reptilian species have long been incorporated into ethnopharmacological practices for their reputed therapeutic benefits [6,7]. For instance, the Chinese blue-tailed skink (*Plestiodon chinensis*) has been traditionally used in Chinese medicine for its diuretic effects, particularly in managing urolithiasis, strangury, and fluid retention [8], typically as the dried whole body after evisceration and administered orally as decoctions or powders. Similarly, the tokay gecko (*Gekko gecko*), a well-known component of Northeast Asian medicine, is also used in the form of the dried whole body and taken either as a decoction or in powdered form. It has shown efficacy in alleviating allergic airway inflammation and mucus hypersecretion through modulation of Th2 cell activation and differentiation [9]. Both *P. chinensis* and *G. gecko* rank among the most widely utilized lizard-derived medicinal resources in traditional Chinese medicine, valued for their therapeutic properties and sustained market demand. Their economic value largely derives from their medicinal use; accordingly, they are described as reptiles with medicinally driven economic value. In response to rising market demand, breeding initiatives for *P. chinensis* and *G. gecko* in China have become progressively standardized, supported by an expanding body of research on captive husbandry and reproductive techniques [10,11,12,13,14]. However, the move toward large-scale reptile farming has also raised concerns about the health and welfare of these captive populations, as intensive rearing practices may increase susceptibility to pathogen transmission and disease outbreaks [15].

Parasitic infections represent one of the most persistent yet often overlooked health challenges in captive reptiles [16,17]. Gastrointestinal parasites such as helminths and protozoans can compromise nutrient uptake, disrupt host immune responses, and elevate the risk of mortality [18]. For instance, Hallinger et al. [19] reported oxyurid nematodes as the most common parasites in captive tortoises, with heavy infestations associated with malabsorption, intestinal impaction, and elevated mortality. In the same study, coinfections with *Balantidium* spp. acted synergistically with bacteria to exacerbate enteritis, and both *Cryptosporidium* spp. and *Balantidium* spp. impaired calcium absorption. However, not all parasitic infections manifest with clinical signs. Amaral et al. [20] observed that leopard geckos (*Eublepharis macularius*) frequently carry intestinal nematodes of the family Oxyuridae and superfamily Strongyloidea without showing clinical signs. While these infections may remain subclinical, they can still compromise host health over time. In cases of chronic or high-burden infestations, reptiles may experience gradual deterioration. Plus, these nematodes have simple direct life cycles, which allow them to complete transmission without intermediate hosts. This biological feature greatly increases the risk of recurrent or rapid reinfection in captive settings, particularly when enclosures lack proper sanitation or husbandry standards. Thus, even seemingly benign infections can become a significant health and welfare issue in intensively managed reptile populations.

Such subclinical infections often go unnoticed until they reach advanced stages, highlighting the critical need for routine parasitological monitoring. In commercial breeding systems, these hidden infections can impair growth rates, diminish product quality, and ultimately lead to substantial economic losses [21,22]. Despite these risks, most surveillance practices continue to rely on conventional microscopy and morphological identification, methods that may overlook mixed or cryptic infections and underestimate the true diversity of parasite communities [23].

Conventional surveillance can miss mixed or low-intensity intestinal infections and underestimate community diversity in captive systems. High-throughput sequencing (HTS) provides a robust alternative, offering higher sensitivity and finer taxonomic resolution in characterizing complex parasite assemblages [24]. By amplifying conserved genetic markers, HTS enables the simultaneous detection of multiple parasite taxa, including those that are otherwise difficult to identify through morphology-based or culture-dependent approaches [25,26]. HTS of 18S rRNA marker genes enables simultaneous detection of helminths and protists at finer taxonomic resolution, improves recognition of coinfections [27], and supports community-level inference relevant to husbandry decisions. Applications across vertebrates [28,29,30,31] show higher breadth and sensitivity than routine microscopy and morphology-based identification and demonstrate the feasibility of fecal metabarcoding for parasite community surveys. While this technique has already proven valuable in livestock and wildlife disease surveillance [32], its application in reptile health assessment particularly within large-scale farming systems of economic importance remains underutilized.

In this study, we employed high-throughput amplicon sequencing to comprehensively profile the intestinal parasite communities of two lizard species of significant medicinal and economic value, *G. gecko* and *P. chinensis*, maintained in captivity. Our approach enabled us to not only characterize the overall community composition but also quantify parasite prevalence across hosts. In addition, we carried out univariable correlation network analyses to identify potential associations between dominant parasitic taxa and co-occurring members of the gut microbiota, offering insights into possible parasite–microbe interactions. By integrating prevalence estimates with interaction networks, our findings provide a more holistic view of parasite dynamics in intensively managed reptiles. Ultimately, this work expands the current understanding of parasite burdens in captive reptile populations and establishes an important reference framework for health monitoring. The insights gained have practical implications for improving surveillance strategies, refining husbandry protocols, and mitigating disease risks, thereby contributing to both animal welfare and the sustainability of reptile farming systems.

## 2. Materials and Methods

### 2.1. Ethical Statement

All experimental procedures involving animals were reviewed and approved by the Institutional Animal Care and Use Committee (IACUC) of Guangxi Normal University (Approval No. 202509-005). All work was conducted in full compliance with the university’s ethical standards as well as internationally recognized guidelines for the care and use of laboratory animals.

### 2.2. Sample Collection

A total of 42 adult lizards, including both males and females, were obtained from licensed breeding facilities in Guangxi, China. The sample comprised Chinese blue-tailed skinks (*P. chinensis*, *n* = 21) from Guilin and tokay geckos (*G. gecko*, *n* = 21) from Nanning. We measured snout vent length (SVL) and weight for each individual, and the corresponding data are provided in Supplementary Table S1. As exact ages were unavailable, we confirmed adult status based on facility records and typical morphological features of mature individuals. All SVL fell within adult size ranges for these species. Upon arrival at the laboratory, all animals were given a one-week acclimation period prior to sampling. Fecal specimens were collected non-invasively through rectal swabbing of live individuals using sterile, single-use sampling swabs (bkmamlab 150102012; Beekman Biotechnology Co., Ltd., Changde, China; swab head diameter 2.5 mm; total length 14.5 cm). Each swab was pre-moistened with sterile saline, carefully inserted just past the cloacal opening into the distal rectum, gently rotated to maximize sample adherence, and then withdrawn. All procedures were carried out by trained personnel under the approved ethical protocol, with measures taken to minimize handling stress and restraint time; anesthesia was not required. Following collection, swabs were immediately stored at −80 °C and subjected to standardized DNA extraction within 24 h to preserve sample integrity.

### 2.3. DNA Extraction and PCR Amplification

DNA extraction and amplicon sequencing were conducted by Majorbio Bio-Pharm Technology Co., Ltd. (Shanghai, China). Total microbial genomic DNA was isolated from fecal samples using the E.Z.N.A.^®^ Soil DNA Kit (Omega Bio-tek, Norcross, GA, USA) following the manufacturer’s instructions. The quality and concentration of the extracted DNA were evaluated using 1.0% agarose gel electrophoresis and a NanoDrop 2000 spectrophotometer (Thermo Scientific, Waltham, MA, USA).

The hypervariable V4 region of the eukaryotic 18S rRNA gene was amplified using the primer pair TAReuk454FWD1F (5′-CCAGCASCYGCGGTAATTCC-3′) and TAReukREV3R (5′-ACTTTCGTTGATYRA-3′) [33] on a T100 Thermal Cycler (Bio-Rad Laboratories, Hercules, CA, USA). Each PCR reaction (20 μL) contained 4 μL of 5× FastPfu Buffer (TransGen Biotech, Beijing, China), 2 μL of 2.5 mM dNTPs, 0.8 μL of each primer (5 μM), 0.4 μL of FastPfu DNA polymerase, approximately 10 ng of template DNA, and nuclease-free water to make up the final volume. The cycling program consisted of an initial denaturation at 95 °C for 3 min, followed by 27 cycles of denaturation at 95 °C for 30 s, annealing at 55 °C for 30 s, and extension at 72 °C for 45 s, with a final extension step at 72 °C for 10 min and a hold at 4 °C. Amplification products were visualized on 2% agarose gels, purified using a PCR Clean-Up Kit (Beyotime Biotechnology, Shanghai, China), and quantified with a Qubit 4.0 fluorometer (Thermo Fisher Scientific, Waltham, MA, USA).

### 2.4. Sequencing and Bioinformatic Processing

Purified amplicons were pooled in equimolar amounts and subjected to paired-end sequencing on the Illumina MiSeq platform (Illumina, San Diego, CA, USA) according to standard protocols provided by Majorbio Bio-Pharm Technology Co., Ltd. (Shanghai, China). After sequencing, raw FASTQ files were demultiplexed with an in-house Perl script using exact barcode matching; primer matching allowed up to two mismatches and read orientation was adjusted accordingly. Quality filtering was performed with fastp (v0.19.6) [34] using a 50 bp sliding window: reads were truncated when the mean quality within the window fell below 20, truncated reads shorter than 50 bp were discarded, and reads containing ambiguous bases were removed. Paired reads were merged with FLASH (v1.2.11) [35] requiring a minimum overlap of 10 bp and a maximum mismatch ratio of 0.2; read pairs that did not meet these criteria were discarded. The merged reads were then denoised with the DADA2 plugin in the QIIME 2 pipeline (v2024.10) [36], with maxEE set to 5 and truncQ set to 0 (no truncation), yielding high resolution amplicon sequence variants at single nucleotide accuracy.

Primary taxonomic classification was performed with the BLAST (v 2.16.0) consensus classifier against the SILVA database (v138) (https://www.arb-silva.de/, accessed on 12 September 2025). Any ASVs with low-confidence assignments or unclassified results were further queried against the NCBI nucleotide (NT) database (https://www.ncbi.nlm.nih.gov/, accessed on 15 September 2025). To identify potential parasitic or pathogenic taxa, ASV-level annotations were cross-referenced with the Pathogen–Host Interactions database (PHI-base, v4.14, https://www.phi-base.org/, accessed on 20 September 2025), and only high-confidence matches were retained for downstream analyses. All raw sequencing data generated in this study have been deposited in the NCBI Sequence Read Archive (SRA) under the accession number PRJNA1333838.

### 2.5. Data Analysis

Rarefaction curves were generated to assess sampling adequacy and compare species richness among samples, following the analytical frameworks described by Mao et al. [37] and Colwell et al. [38]. Curve construction was performed in mothur (v1.30) [39] with 1000 iterations per sample. These curves illustrate the relationship between sequencing depth and the rate of ASV detection, thereby allowing evaluation of whether sequencing effort sufficiently captured the underlying community diversity.

Multivariable statistical analyses and ordination were carried out in R (v4.3.3) using the vegan package. To address data sparsity and reduce the impact of zero inflation, community composition data were subjected to Hellinger transformation, as recommended for ecological datasets [40]. Principal component analysis (PCA) was then applied to visualize host-level separation, with taxa loadings used to interpret contributions of specific groups. Statistical significance of group differences was tested using Hotelling’s T^2^ test [41] and permutational multivariable analysis of variance (PERMANOVA, 999 permutations) [42]. In addition, univariate tests on PCA axes were performed, including the Mann–Whitney U (MWU) and Welch’s t test, with effect size estimated by Cliff’s Δ. To determine whether observed differences were due to variation in dispersion or actual centroid separation, homogeneity of dispersion was evaluated using PERMDISP [43].

To explore potential co-occurrence patterns between dominant microbial and parasitic genera in captive reptiles, we conducted univariable correlation network analysis in Python (v2.7.0) using the stat module. Genus-level relative abundances were used to calculate Spearman’s rank correlation coefficients, focusing on the 50 most abundant genera. Only correlations with absolute coefficient values (|ρ|) ≥ 0.6 and statistical significance at *p* < 0.05 were retained for network construction. Putative pathogenic parasites were identified by cross-referencing ASV-level taxonomic annotations with known pathogenic genera. For each host species, any sample containing at least one ASV assigned to a pathogenic genus was classified as parasite-positive. The number of positive individuals and their associated parasite taxa were recorded, and infection proportions were calculated as the proportion of infected individuals relative to the total number of hosts sampled. To quantify the uncertainty around the estimated infection proportions and to facilitate fair comparison across groups, we calculated two sided 95 percent confidence intervals for binomial proportions [44] using the Wilson score method [45] and report all intervals to one decimal place, including categories with zero positives. Detailed values are provided in Supplementary Table S2. Comparisons of infection proportions between host species, as well as between sexes within species, were performed using two-sided Fisher’s exact tests. To control for multiple testing across parasite genera, *p*-values were adjusted using the Benjamini–Hochberg false discovery rate (FDR) method. Odds ratios and corresponding 95% confidence intervals were estimated with the Haldane–Anscombe continuity correction to account for contingency tables with zero counts.

## 3. Results

### 3.1. ASV-Based Rarefaction Patterns

**Figure 1:**
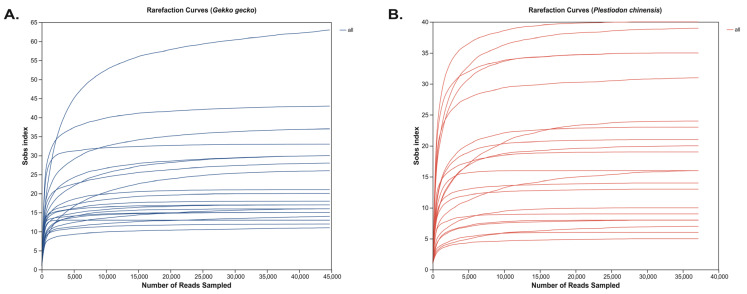
Sample-based rarefaction curves of observed eukaryotic richness (Sobs index) in fecal libraries from captive reptiles. (**A**) represents *Gekko gecko* and (**B**) represents *Plestiodon chinensis*.

Rarefaction curves generated from ASV-level taxonomic profiles displayed a steep initial increase in detected taxa, followed by a plateau, suggesting that the applied sequencing depth was sufficient to capture the majority of microbial diversity (Figure 1). This trajectory is characteristic of adequately sampled communities and suggests that the sequencing effort captured the majority of the underlying microbial and parasitic diversity present in the samples. Both *G. gecko* and *P. chinensis* samples displayed asymptotic rarefaction curves, indicating that their microbial communities were sampled to near saturation. Importantly, this plateauing trend was consistently observed across individual samples within each species, further reinforcing that sequencing coverage was sufficient and not biased by host-specific variability. The convergence of curves across samples provides strong evidence that the observed differences in microbial and parasitic assemblages reflect true biological variation rather than undersampling artifacts. Thus, the sequencing depth employed not only ensures reliable estimation of alpha diversity metrics but also underpins the robustness of downstream comparative and multivariable analyses.

### 3.2. Host Separation Revealed by PCA

**Figure 2:**
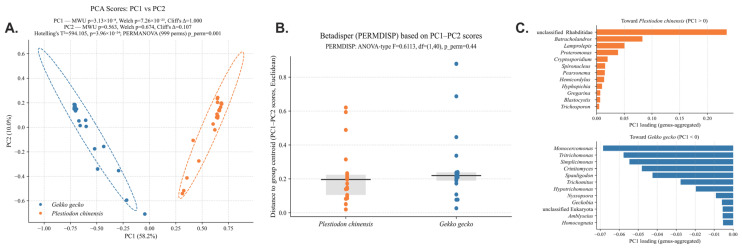
Differences in fecal eukaryotic communities between host species. (**A**) PCA of genus-level pro-files shows clear separation between *Gekko gecko* and *Plestiodon chinensis* (PERMANOVA *p* = 0.001). (**B**) Community dispersion does not differ significantly between hosts (PERMDISP: F = 0.6113, df = (1,40), *p* = 0.44). (**C**) PC1 loadings highlight the taxa contributing most strongly to the observed between-host separation.

Principal component analysis (PCA) based on ASV-level profiles (*n* = 21 per host species) revealed a distinct separation of parasite communities between the two lizard hosts (Figure 2A). The first principal component (PC1), which explained 58.2% of the total variance, differentiated *P. chinensis* (positive PC1 scores) from *G. gecko* (negative PC1 scores), while the second principal component (PC2) accounted for an additional 10.0% of the variance. Multivariable statistical testing confirmed this strong host-associated divergence: Hotelling’s T^2^ test (T^2^ = 594.105, F = 289.6262, *p* = 3.96 × 10^−24^) and PERMANOVA (F = 156.2054, *p* = 0.001, 999 permutations) both demonstrated highly significant differences between groups. In addition, univariable tests on PCA axes showed that PC1 differed markedly between hosts (MWU *p* = 3.13 × 10^−8^, Welch *p* = 7.26 × 10^−23^, Cliff’s Δ = 1.000), whereas PC2 showed no significant difference (MWU *p* = 0.563, Welch *p* = 0.674, Cliff’s Δ = 0.107). Dispersion analysis (PERMDISP) indicated that variance structure did not differ significantly (ANOVA-type F = 0.6113, *p* = 0.44) (Figure 2B). As is shown in Figure 2C, the *P. chinensis* group exhibited a relatively balanced parasite assemblage, comprising nematodes, protists, and yeasts. The most influential contributor was an unclassified Rhabditidae lineage (PC1 loading = 0.235), suggesting that nematode dominance strongly shaped the parasite community in skinks. Other taxa positively associated with *P. chinensis* included *Cryptosporidium*, *Gregarina*, *Proteromonas*, *Spironucleus*, and two yeast genera (*Hyphopichia* and *Trichosporon*), reflecting a diverse and complex parasitic and microbial ecosystem. By contrast, *G. gecko* was characterized by strong enrichment of trichomonads, including *Hypotrichomonas*, *Monocercomonas*, *Simplicimonas*, *Tritrichomonas*, and *Trichomitus*, which collectively dominated its parasite community. Additional contributions came from nematodes such as *Spauligodon*, as well as the yeast *Crinitomyces*. The predominance of trichomonads in *G. gecko* suggests a host-specific predisposition toward colonization by flagellated protists, possibly linked to host gut physiology, immune responses, or husbandry conditions. These findings demonstrate clear host-specific structuring of parasite communities in captive reptiles, with *P. chinensis* harboring a more diverse and balanced assemblage of nematodes, protists, and yeasts, while *G. gecko* was dominated by trichomonads. The significant separation confirmed by multivariable tests indicates that parasite composition is strongly influenced by host identity rather than random variation. Despite comparable within-group dispersion across hosts (PERMDISP, *p* > 0.05), the balanced co-occurrence of nematodes, protists, and yeasts in *P. chinensis* may suggest higher susceptibility to complex co-infections, whereas the trichomonad dominance in *G. gecko* may predispose this species to protist-driven gastrointestinal disorders.

### 3.3. Parasite Infection Patterns

**Figure 3:**
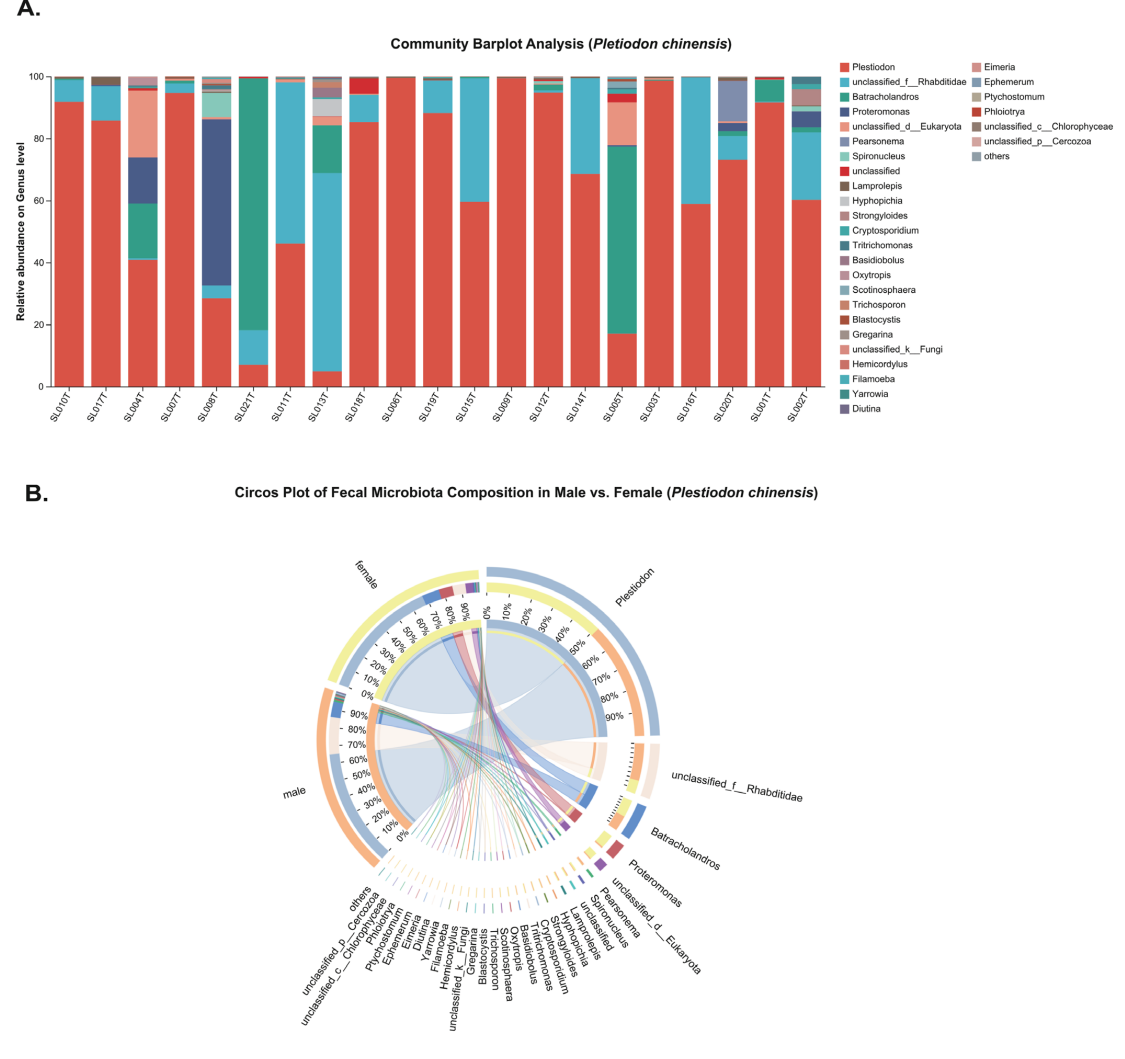
Genus-level composition of fecal eukaryotic communities in *Plestiodon chinensis*. (**A**) Stacked bar plots displaying genus-level relative abundances for each individual sample. (**B**) Circos plot illustrating comparisons between males and females, with ribbon widths representing rescaled genus-level relative abundance percentages.

**Figure 4:**
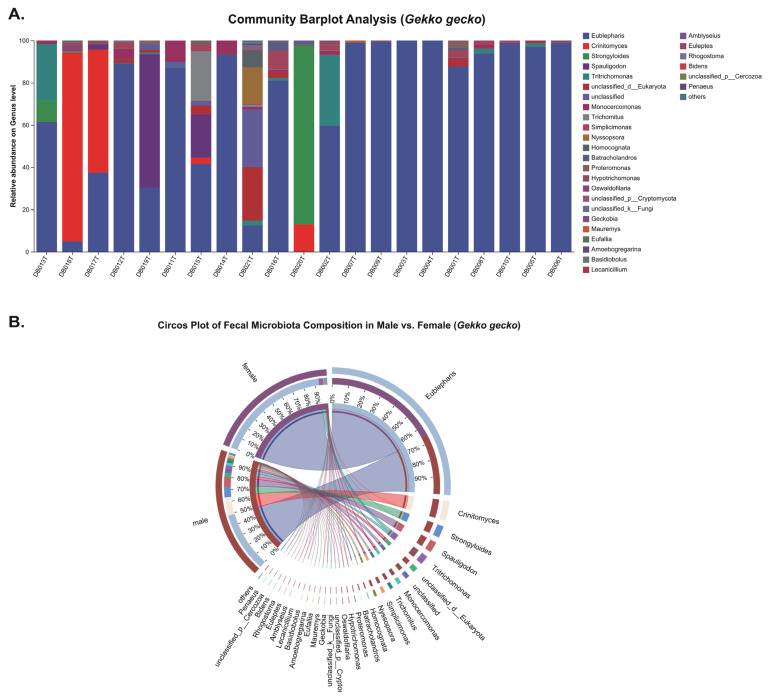
Genus-level composition of fecal eukaryotic communities in *Gekko gecko*. (**A**) Stacked bar plots showing genus-level relative abundances in individual samples. (**B**) Circos plot comparing males and females, with ribbon widths representing rescaled genus-level relative abundance percentages.

In *P. chinensis*, the genus-level bar plot (Figure 3A) reveals the occurrence of four pathogenic parasite genera: *Cryptosporidium*, *Eimeria*, *Strongyloides*, and *Spironucleus* detected in sampled individuals. In contrast, *G. gecko* harbored *Oswaldofilaria, Strongyloides*, *Spauligodon* and *Spironucleus* (Figure 4A). The circos plots (Figure 3B and Figure 4B) illustrate associations between parasite genera and host sex, indicating that these taxa were present in both male and female individuals, with no clear sex-specific patterns of occurrence.

Among the 21 sampled individuals of each host species, the overall parasite infection proportion was 42.8% (9/21) in *G. gecko* and 57.1% (12/21) in *P. chinensis* (Table 1), with no statistically significant difference between the two hosts (Fisher’s exact *p* = 0.5377, q = 0.941; OR = 0.58, 95% CI: 0.18–1.90). In *G. gecko*, the infection proportions were 4.8% (1/21) for *Oswaldofilaria*, 19.1% (4/21) for *Strongyloides*, 14.3% (3/21) for *Spauligodon*, and 9.5% (2/21) for *Spironucleus* while *Cryptosporidium* and *Eimeria* were absent. Conversely, in *P. chinensis*, *Cryptosporidium* was the most prevalent parasite, detected in 57.1% (12/21) of individuals, followed by 14.3% (3/21) for *Eimeria*, *Strongyloides*, and *Spironucleus*; *Oswaldofilaria* and *Spauligodon* were not detected. A statistically significant host-specific difference was observed only for *Cryptosporidium*, which was exclusively found in *P. chinensis* (0/21 vs. 12/21; *p* = 5.32 × 10^−5^, q = 0.0003; OR = 0.018, 95% CI: 0.001–0.330).

Sex-associated patterns of parasite infection were examined separately for each host species (Table 2). In *G. gecko*, *Strongyloides* was detected at comparable frequencies in females (20.0%, 2/10) and males (18.2%, 2/11). *Spauligodon* occurred exclusively in males (27.3%, 3/11), while *Oswaldofilaria* (absent in females; 9.1%, 1/11 in males) and *Spironucleus* (10.0%, 1/10 in females; 9.1%, 1/11 in males) showed low prevalence without clear sex bias. In *P. chinensis*, *Cryptosporidium* was found in both sexes at similar rates (55.6%, 5/9 in females; 58.3%, 7/12 in males). The other genera (*Eimeria*, *Strongyloides* and *Spironucleus*) showed moderate infection levels, ranging from 11.1% (1/9) to 16.7% (2/12) across both sexes. No statistically significant differences were observed for any parasite genus either between the two host species or between males and females within species, as all FDR-adjusted q-values exceeded 0.05. Relative abundance patterns showed only minor variation between sexes, with no consistent directional trends. The findings indicate that both *G. gecko* and *P. chinensis* carry multiple intestinal parasites, but without notable sex-related differences. A key finding is the exclusive and high prevalence of *Cryptosporidium* in *P. chinensis*, suggesting species-specific susceptibility. These results emphasize host species as the main determinant of parasite patterns and provide important baseline data for captive reptile health management and future ecological studies.

### 3.4. Parasite–Microbe Co-Occurrence Network

**Figure 5:**
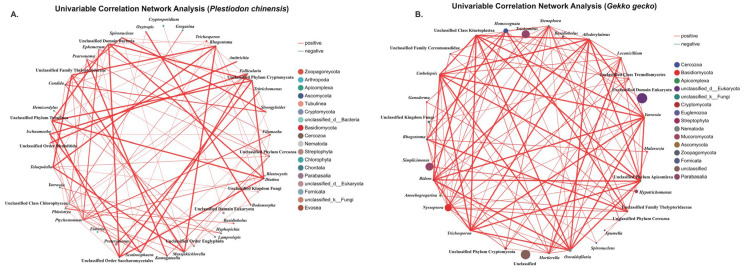
Univariable correlation network analysis of parasite–microbe associations within fecal eukaryotic communities. Nodes correspond to parasite genera and associated eukaryotic taxa, while edges represent significant positive Spearman correlations (*p* < 0.01). Edge width is scaled to correlation strength (ρ), node color indicates taxonomic group, and node size reflects node degree. Panels (**A**,**B**) show the networks for *Plestiodon chinensis* and *Gekko gecko*, respectively.

Univariable correlation analysis identified strong positive associations between intestinal parasites and a range of eukaryotic microbes in both *G. gecko* and *P. chinensis* (Figure 5). Pathogenic taxa such as *Cryptosporidium*, *Eimeria*, *Oswaldofilaria*, *Strongyloides*, *Spauligodon*, and *Spironucleus*, showed significant correlations with fungal genera including *Candida*, *Lecanicillium*, and *Mortierella*, as well as protists such as *Homocognata* and *Rhogostoma*. The host stratified Spearman networks at the genus level (|ρ| ≥ 0.60 and *p* < 0.05) contained 31 nodes and 109 edges for *G. gecko* and 43 nodes and 90 edges for *P. chinensis*. To aid interpretation, the univariable correlation network emphasizes associations among dominant genera rather than all detected taxa. Spearman’s correlation coefficients ranged from 0.606 to 0.997, with all associations significant at *p* < 0.01 (Table 3). These results indicate stable co-occurrence patterns that may reflect underlying ecological or functional interactions within the reptile gut. The significance of these findings lies in their potential to reveal how parasites interact with other microbial taxa, suggesting that co-infections or microbial shifts could influence parasite persistence, host susceptibility, and overall gut health, thereby underscoring the need to consider parasite–microbe interactions in reptile health management.

## 4. Discussion

Intestinal parasitism is a well-recognized challenge in animal production systems, where it reduces feed efficiency, slows growth, and increases mortality [46,47,48,49], ultimately causing significant economic losses. In industries such as aquaculture and livestock farming, these impacts are well documented [50,51,52], and parasite control forms a core aspect of production management.

In contrast, the economic consequences of parasitic infections in captive reptiles remain underexplored. This is particularly important for intensively farmed species of high economic or medicinal value, where parasite-related health declines can translate into hidden financial costs [53]. Such infections may impair weight gain, delay development, increase reliance on veterinary care, and undermine the profitability of farming operations. These risks are often overlooked in current practices, underscoring the need for systematic monitoring and targeted control measures.

Captive reptiles face growing exposure to a wide variety of parasitic infections, with husbandry conditions and feeding practices playing a central role in promoting transmission [54]. Amaral et al. [20] highlighted that poor hygiene and contaminated food sources were key drivers of parasite persistence in captive *E. macularius*. Dantas et al. [17] observed that reptiles in captivity often carry heavy parasite loads, largely due to the closed, high-density, and contamination-prone nature of these environments, which enable continuous circulation of parasites. This risk is further heightened because many reptile parasites have simple direct life cycles or are easily transmitted through the consumption of infected prey or food items.

In this study, we detected multiple pathogenic parasitic genera, such as *Cryptosporidium*, *Eimeria*, *Strongyloides*, *Spauligodon*, *Spironucleus* and *Oswaldofilaria*, in captive *P. chinensis* and *G. gecko*. Many of these are well-known agents of clinical disease in reptiles [16,55,56,57], and some also pose zoonotic risks [58], underscoring the importance of regular surveillance and improved management practices in captive settings. Despite the absence of statistically significant differences in overall parasite infection proportion between host species or sexes, the observed variation in community composition and coinfection patterns likely reflects underlying ecological and epidemiological nuances. For instance, coinfections involving *Cryptosporidium* were more frequently observed in *P. chinensis*, possibly reflecting species-specific susceptibility; this parasite accounted for a large proportion of infections in *P. chinensis*, thereby elevating the overall infection proportion. In contrast, although coinfections were less frequent in *G. gecko*, parasite richness was slightly higher, which also contributed to an elevated infection proportion. These complementary patterns may explain why overall prevalence did not differ significantly between hosts despite divergent community structure. Furthermore, the lack of sex-specific differences in parasite infection proportions within host species may reflect comparable immunological susceptibility between males and females under standardized captive conditions, where sex-related variation in immune function may be reduced.

A notable finding was the significant interspecific difference in *Cryptosporidium* prevalence (*p* = 5.32 × 10^−5^, Fisher’s exact test), with a high infection proportion in *P. chinensis* (57.14%, 12/21) and complete absence in *G. gecko*. This striking contrast may indicate host-specific resistance to *Cryptosporidium*. Previous research has shown that reptile taxa differ widely in their susceptibility to this parasite [59,60], potentially influenced by species-specific factors such as immune responses, gut microbiome composition, or epithelial receptor characteristics. It is possible that *G. gecko* possesses greater resistance to intestinal colonization or replication by *Cryptosporidium*, limiting the establishment of infection even when exposed. Alternatively, environmental or husbandry-related differences may have influenced the observed variation. Although both species were bred in captivity, factors such as housing conditions, hygiene standards, enclosure density, and feeding practices at different breeding facilities could affect exposure levels and transmission risk. Poor sanitation and overcrowding, in particular, have been consistently linked to *Cryptosporidium* outbreaks in animals [61,62]. Thus, the distinct infection patterns observed are likely shaped by a combination of host-specific physiological defenses and management practices.

*Cryptosporidium* is a protozoan parasite that poses serious health risks to a wide range of reptilian hosts. While disease severity varies with both the reptile species and the infecting *Cryptosporidium* strain, a hallmark of infection is the onset of severe gastrointestinal disease [60]. Its zoonotic capacity further elevates the public health concerns associated with infection [63,64]. In snakes, *Cryptosporidium* commonly causes marked gastrointestinal disorders, including mid-body abdominal swelling, anorexia, regurgitation, and weight loss [65]. In *E. macularius*, infection manifests as chronic weight loss, diarrhea, anorexia, cachexia, and lethargy, with juveniles being particularly vulnerable and often experiencing high mortality rates [66]. Transmission typically occurs via ingestion of contaminated food or water, or indirectly through fomites such as hands, enclosures, or feeding equipment contaminated with oocysts [67]. In captive environments, insufficient cleaning of substrates and water systems enables oocysts to persist for long periods, supporting recurrent infections [68]. Furthermore, cross-contamination readily arises among reptiles kept in shared enclosures or handled with the same husbandry tools [69].

On the other hand, host–parasite coevolution has likely driven the adaptive evolution of reptilian immune mechanisms such as pattern recognition receptors (PRRs) and downstream signaling pathways. This interpretation is consistent with functional divergence of reptilian PRRs and signatures of positive selection across TLR/RLR/NLR families in reptiles [70,71,72]. However, immune regulation that limits host tissue damage, along with epitope masking effects mediated by natural antibodies, may inadvertently create favorable conditions for parasite persistence or immune evasion [73]. Evidence from chelonians [74,75] shows trade-offs between natural and acquired antibody responses and putative epitope masking, aligning with this mechanism. Our study revealed a significant disparity in *Cryptosporidium* prevalence between *P. chinensis* and *G. gecko*, which may suggest that host-specific differences in the sensitivity of PRRs pathways or immune regulatory thresholds underlie variation in susceptibility. But this hypothesis warrants targeted investigation using comparative transcriptomic profiling or receptor–ligand binding assays. Beyond the effects of individual pathogens, coinfections warrant special consideration due to their complex and multifactorial impacts on host pathology. Interactions between parasites and other pathogens can intensify disease severity, heighten vulnerability to secondary infections, complicate diagnosis, and reduce vaccine effectiveness. These outcomes often translate into heavier pathogen burdens, more severe clinical signs, greater tissue and organ damage, and disrupted immune responses [76]. In reptiles, such coinfection-driven dynamics are increasingly documented in captivity. For instance, farmed tortoises in southern Italy show high rates of both Salmonella and oxyurid infections, with a significant positive association between the two pathogens [77]. In captive snakes, *Cryptosporidium* acts as a primary lethal pathogen in coinfected individuals, while microsporidia function opportunistically, exploiting the host’s compromised state [78]. Similarly, simultaneous infections with multiple parasites can promote each other’s persistence, aggravate immune exhaustion, hinder nutrient uptake, and cause extensive tissue or organ damage [79]. Guardone et al. [80] reported that inland bearded dragons (*Pogona vitticeps*) frequently harbor multiple parasitic infections, with both single and mixed infections strongly linked to clinical disease. In our study, although *G. gecko* and *P. chinensis* showed comparable overall infection proportions (42.86%, 9/21 vs. 57.14%, 12/21), multiple *P. chinensis* individuals carried coinfections involving *Cryptosporidium* together with one or more additional genera, including *Eimeria*, *Strongyloides*, and *Spironucleus* (Figure 3A). In this study, coinfection with *Strongyloides* and *Spironucleus* was detected in individuals of both host species. A strong positive correlation between the two genera (Spearman ρ = 0.9965, *p* = 6.06 × 10^−22^) suggests that their co-occurrence may arise from synergistic pathogenic interactions or overlapping strategies for host exploitation. Similar patterns have been noted in amphibians, where *Strongyloides* infections are often accompanied by intestinal flagellates of the genus *Spironucleus*, with such combinations linked to severe gastrointestinal disease. Hallinger et al. [57] further demonstrated that coinfection with *Spironucleus* and nematodes in amphibians can result in fatal enteritis. In contrast, only one *G. gecko* individual harbored coinfection, with most infections limited to a single parasite genus (Figure 4A). Interestingly, parasite interactions during coinfection are not always synergistic and can also be antagonistic. For example, in an in vitro chicken macrophage model, simultaneous infection with *Eimeria* and *Cryptosporidium* led to mutual inhibition of intracellular replication [81], which may partially explain the lower prevalence of *Eimeria* compared to *Cryptosporidium* observed in the present study. Conversely, we observed coinfections involving *Eimeria* and intestinal helminths. Such combinations are known to act synergistically, causing significant intestinal mucosal damage, weakening epithelial barrier function, and eliciting more severe inflammatory responses and tissue injury [82]. Such interactions can also compromise host immune defenses, heightening vulnerability to secondary infections and contributing to productivity losses, thereby underscoring the need for closer consideration in management practices. The detection of *Spironucleus* in this study is particularly significant, as this genus has been linked to infectious catarrhal enteritis in commercially farmed chukar partridges (*Alectoris chukar*). In these birds, coinfection with additional pathogens has been shown to greatly intensify clinical severity and tissue damage, resulting in higher mortality, aggravated diarrhea, lethargy, and characteristic intestinal lesions, including villus blunting, villus fusion, and pronounced mucosal inflammation [83].

Coinfections involving eukaryotic parasites and other microbes are commonly observed in captive systems and may exert either synergistic or antagonistic effects on host health. Evidence from managed populations illustrates this duality: in a captive anaconda, concurrent infection with *Entamoeba invadens* and *Aeromonas hydrophila* resulted in necrotizing gastroenteritis and hepatocellular necrosis [84], while in an Aldabra giant tortoise (*Geochelone gigantea*), colonic ulcers caused by *Candida* were accompanied by secondary protozoan infection [85]. Experimental studies in poultry further demonstrate these dynamics coinfection with *Eimeria acervulina* and *E. tenella* impaired growth and worsened intestinal [86], whereas dual infection with *Histomonas meleagridis*, *Heterakis gallinarum*, and *Ascaridia galli* showed largely exclusive excretion patterns, suggesting antagonistic interactions [87].

To investigate potential interactions between parasites and other members of the host-associated eukaryotic microbiota, we conducted a univariable correlation analysis at the genus level. *Oswaldofilaria*, a filarial parasite known to cause nodular lesions on internal organs, subcutaneous tissues, and musculature of reptilian hosts [56]. Transmission typically involves mosquitoes serving as intermediate hosts [88]. In our study, *Oswaldofilaria* was detected in only one *G. gecko* individual (4.76%, 1/21), indicating a low prevalence in the sampled population. Interestingly, this parasite exhibited a significant positive correlation with the fungal genus *Lecanicillium* (Spearman ρ = 0.72457, *p* = 2.03 × 10^−4^). Notably, *Lecanicillium* infect insects, including mosquitoes, which are vectors of filarial worms, and are being investigated as biopesticides for controlling filarial transmission. Kataki et al. [89] demonstrated the pathogenic potential of *Lecanicillium lecanii* against Culex mosquitoes, highlighting its possible use in reducing filarial transmission. More recently, *Lecanicillium* fungi have also been reported for the first time as reptile pathogens, causing both cutaneous and systemic mycoses in lizards [90]. However, the observed positive correlation between *Lecanicillium* and *Oswaldofilaria* in *G. gecko* may indicate a potential biological link. *Lecanicillium* species are known to infect mosquitoes that serve as intermediate hosts for *Oswaldofilaria*. This known association suggests that mosquito-mediated interactions could play a role in their co-occurrence. Nevertheless, the specific mechanism underlying this relationship remains uncertain and requires further investigation. In contrast, coinfections involving *Spironucleus* and other flagellated protists such as *Tritrichomonas* have been shown to act synergistically in aggravating enteric disease. In amphibians, concurrent infections with multiple protozoan parasites, including *Spironucleus* and *Tritrichomonas*, have been associated with severe and often fatal enteritis [57]. In line with these observations, our univariable correlation analysis identified a significant positive association between *Spironucleus* and *Tritrichomonas* in *P. chinensis* (Spearman ρ = 0.62481, *p* = 2.46 × 10^−3^), suggesting a possible cooperative interaction that could intensify gastrointestinal disease in coinfected hosts. Additionally, in *P. chinensis*, *Candida* abundance showed strong positive correlations with both *Strongyloides* (Spearman ρ = 0.83697, *p* = 2.24 × 10^−6^) and *Spironucleus* (Spearman ρ = 0.83279, *p* = 2.81 × 10^−6^). These correlations indicate that infections with *Strongyloides* or *Spironucleus* may facilitate conditions that promote the overgrowth of *Candida*. Possible mechanisms include physical disruption of the intestinal mucosa through parasite feeding or attachment, which compromises epithelial integrity, as well as changes in nutrient availability in the gut that favor fungal colonization [91,92]. Previous studies have shown that reptiles are particularly vulnerable to candidiasis when immune defenses are impaired or when the intestinal barrier is weakened [85,93,94,95]. Under such circumstances, *Candida* can shift from a commensal to a pathogenic state, leading to enteritis, systemic mycoses, or secondary complications. Thus, the strong positive associations observed here may reflect underlying synergistic interactions, where parasitic infections not only damage host tissues but also create an ecological niche that enables fungal proliferation, ultimately exacerbating host morbidity. In addition, it is important to recognize that *Strongyloides* exhibits a complex life cycle involving both parasitic and free-living stages [96]. Inadequate substrate management may result in persistently moist conditions within captive enclosures, which could allow *Strongyloides* to complete its free-living cycle entirely within the breeding facility [97], thereby facilitating continuous transmission. Wet conditions in the enclosures may also contribute to fungal proliferation, creating an ecological overlap that promotes co-occurrence [98,99]. Proper husbandry practices are essential for minimizing enteric pathogen loads and disrupting transmission cycles in captive reptile populations. Enclosures should be designed to provide sufficient space, species-appropriate temperature and humidity gradients, suitable photoperiods with UVB exposure, and clean, easily replaceable substrates [100]. Water sources for both drinking and bathing must be regularly refreshed and disinfected, while strict use of enclosure-specific tools and gloves is necessary to reduce the risk of fomite-mediated transmission [101]. Maintaining biologically appropriate stocking densities is crucial, as overcrowding can heighten stress, weaken immune defenses, and accelerate pathogen spread. Decisions regarding co-housing should carefully consider species compatibility, behavioral interactions, and relative disease risks to prevent unnecessary exposure to parasites or pathogens [15]. Quarantine and isolation procedures should be rigorously enforced for new or symptomatic animals, and facility layouts should support unidirectional workflows to reduce cross-contamination [102]. Equally important is comprehensive personnel training, ensuring that staff understand and consistently follow biosecurity protocols. This includes sequencing animal handling from healthy to potentially infected individuals, proper use of protective clothing and disposable materials, and rigorous disinfection of equipment and enclosures between uses. Finally, commercial production systems should implement continuous and systematic pathogen surveillance, supported by routine diagnostic testing, to enable early detection of infections and rapid intervention before outbreaks escalate. Together, these measures provide an integrated framework for safeguarding animal health, improving productivity, and reducing economic losses in captive reptile operations.

In commercial and intensively farmed captive animal systems, a range of diagnostic approaches are routinely used for parasite surveillance and control. Among these, direct fecal smears examined under light microscopy remain the primary frontline method, valued for their speed and simplicity in routine screening [103,104]. This method is commonly supplemented with additional techniques to improve diagnostic accuracy [16]. For detecting low-intensity or intermittent infections, sedimentation and formalin–ethyl acetate stool concentration techniques are particularly useful, as they increase the recovery of protistan cysts, trophozoites, and helminth eggs or larvae [105]. Comprehensive diagnosis is best achieved through a combination of microscopy, necropsy, and real-time PCR (rtPCR) [106]. While microscopy provides rapid morphological identification, rtPCR greatly improves sensitivity and enables species-level resolution. Beyond direct detection, long-term monitoring of body weight and body condition also serves as a valuable indirect indicator of parasite burden in captive reptiles [80]. Collectively, these integrated strategies offer a robust framework for early detection, timely intervention, and effective health management in ex situ reptile populations.

Our findings offer an important snapshot of intestinal parasite communities in two commonly bred reptile species, revealing both host-specific differences and notable co-occurrence patterns that may hold biological significance. While the study was constrained by a relatively small sample size and the absence of detailed environmental records such as enclosure conditions, it nonetheless provides meaningful baseline information on parasite diversity in captive breeding systems. Like other amplicon-based high-throughput sequencing approaches, our method may miss detection of rare or difficult to amplify taxa and is subject to compositional bias from sequencing depth variability. In addition, the cross-sectional sampling design limits inference on infection dynamics, seasonal fluctuations, or turnover events that may occur over time. Age stratification was not performed because individual ages were not available; however, we verified adult status based on breeder records and diagnostic morphological features, and snout–vent lengths were within the expected adult range. Detailed health scoring was not systematically recorded, but all animals appeared phenotypically healthy, exhibited normal feeding and defecation behavior, and showed no signs of emaciation. Even in the presence of relatively high infection proportions and mixed infections in hosts, this may reflect that parasite burdens did not exceed pathogenic thresholds, or that the infecting taxa exhibited low overall virulence. Moreover, in nutritionally adequate adult lizards, host tolerance mechanisms may suppress pathology without necessarily reducing parasite loads, leading to high prevalence but minimal clinical signs. These limitations notwithstanding, our findings offer a valuable foundation for future surveillance and hypothesis-driven research. We underscore the potential influence of both host physiology and management practices on parasite prevalence and interactions. To build on these insights, future research should include broader geographic sampling across multiple breeding facilities, integration of controlled environmental and husbandry data, and longitudinal monitoring of parasite dynamics over time. Such approaches will not only clarify the mechanisms driving parasite transmission but also support the development of more effective surveillance, biosecurity, and management strategies tailored to captive reptile populations.

## 5. Conclusions

This study employed high-throughput sequencing to uncover distinct intestinal parasite infection profiles in two economically and medicinally important lizard species, the Chinese blue-tailed skink (*P. chinensis*) and the tokay gecko (*G. gecko*), maintained under captive conditions. The results demonstrated clear host-specific differences in parasite assemblages, highlighting that each species harbors unique communities shaped by both nematodes and protists, with varying degrees of diversity and dominance. Moreover, the observed significant correlations between parasitic taxa and diverse gut microbes suggest the presence of intricate ecological interactions, where co-occurring fungi and protists may influence parasite persistence, host susceptibility, and overall gut health. The significance of these findings extends beyond basic parasitology, as they underscore the importance of integrating parasite surveillance with microbiota studies to better understand the multifaceted drivers of disease risk in captive reptiles. From a practical standpoint, these insights provide a foundation for health management in reptile farming, such as tailoring deworming protocols to specific parasite profiles or modifying feeding and sanitation regimes to limit microbiota-mediated facilitation of infection. Such applications could help mitigate parasite-related economic losses and improve overall animal welfare in commercial breeding operations. Looking ahead, future research should expand geographic sampling to capture broader population-level variation, incorporate controlled experiments to disentangle the effects of environmental and husbandry conditions, and apply longitudinal monitoring to track temporal dynamics of infection. Such approaches will be critical to elucidating transmission pathways, identifying key risk factors, and unraveling the mechanistic underpinnings of parasite–microbiota interactions, ultimately contributing to improved sustainability and biosecurity in reptile breeding systems.

## Figures and Tables

**Table 1:** Infection proportions of various parasite genera identified in two captive reptile hosts.

Host	Infection Proportion(s) (%)	Parasite Infection Proportion(s) (%)					
		*Cryptosporidium * ^ 1^	*Eimeria*	*Oswaldofilaria*	*Strongyloides*	*Spauligodon*	*Spironucleus*
*Gekko gecko*	42.9% (9/21)	-	-	4.8% (1/21)	19.1% (4/21)	14.3% (3/21)	9.5% (2/21)
*Plestiodon chinensis*	57.1% (12/21)	57.1% (12/21)	14.3% (3/21)	-	14.3% (3/21)	-	14.3% (3/21)

^1^ statistically significant differences (*p* < 0.05) in infection proportions between host species as determined by a two-tailed Fisher’s exact test.

**Table 2:** Parasite infection proportions in *Gekko gecko* and *Plestiodon chinensis*, showing both overall prevalence and sex-specific rates for each parasite genus.

Host	Parasite Genus	Infection Proportion(s) (%)	Infection Proportion(s) in Female (%)	Infection Proportion(s) in Male (%)
*Gekko gecko*	*Oswaldofilaria*	4.8% (1/21)	0.00%	9.1% (1/11)
	*Strongyloides*	19.1% (4/21)	20.0% (2/10)	18.2% (2/11)
	*Spauligodon*	14.3% (3/21)	0.00%	27.3% (3/11)
	*Spironucleus*	9.5% (2/21)	10.0% (1/10)	9.1% (1/11)
*Plestiodon chinensis*	*Cryptosporidium*	57.1% (12/21)	55.6% (5/9)	58.3% (7/12)
	*Eimeria*	14.3% (3/21)	11.1% (1/9)	16.7% (2/12)
	*Strongyloides*	14.3% (3/21)	11.1% (1/9)	16.7% (2/12)
	*Spironucleus*	14.3% (3/21)	11.1% (1/9)	16.7% (2/12)

**Table 3:** Significant positive correlations between parasite genera and associated eukaryotic taxa in *Gekko gecko* and *Plestiodon chinensis*, as determined by Spearman’s rank correlation (ρ) with corresponding *p*-values. Only associations with *p* < 0.01 are presented.

Host	Parasite Genus	Associated Taxon/Parasite	Spearman ρ	*p*-Value
*Gekko gecko*	*Oswaldofilaria*	*Nyssopsora*	0.65211	1.36 × 10^−3^
		*Stenophora*	0.65211	1.36 × 10^−3^
		*Lecanicillium*	0.72457	2.03 × 10^−4^
		*Mortierella*	0.72457	2.03 × 10^−4^
	*Spironucleus*	*Homocognata*	0.65211	1.36 × 10^−3^
		family Thelypteridaceae	0.65211	1.36 × 10^−3^
		*Rhogostoma*	0.65211	1.36 × 10^−3^
*Plestiodon chinensis*	*Cryptosporidium*	unclassified Eukaryota	0.6138	3.08 × 10^−3^
	*Eimeria*	*Oxytropis*	0.60645	3.56 × 10^−3^
		*Bodomorpha*	0.60645	3.56 × 10^−3^
	*Spironucleus*	*Candida*	0.83279	2.81 × 10^−6^
		*Tritrichomonas*	0.62481	2.46 × 10^−3^
		*Rhogostoma*	0.60645	3.56 × 10^−3^
		*Massjukichlorella*	0.60645	3.56 × 10^−3^
		family Thelypteridaceae	0.60645	3.56 × 10^−3^
		*Telaepolella*	0.60645	3.56 × 10^−3^
	*Strongyloides*	*Tritrichomonas*	0.62725	2.34 × 10^−3^
		*Spironucleus*	0.9965	6.06 × 10^−22^
		*Candida*	0.83697	2.24 × 10^−6^

## Data Availability

All raw sequence data generated in this study have been deposited in the NCBI Sequence Read Archive (SRA) under accession number PRJNA1333838.

## Supplementary Materials

The following supporting information can be downloaded at: https://www.mdpi.com/article/10.3390/ani15223298/s1, Table S1: Host morphometrics and sex information; Table S2: Estimated infection proportions and Wilson 95% confidence intervals by host, parasite, and group.
